# Encouraging rational antibiotic prescribing behaviour in primary care – prescribing practice among children aged 0–4 years 2016–2018: an observational study

**DOI:** 10.1080/02813432.2021.1958506

**Published:** 2021-08-04

**Authors:** Maria Run Gunnlaugsdottir, Kristjan Linnet, Jon Steinar Jonsson, Anna Bryndis Blondal

**Affiliations:** a Faculty of Pharmaceutical Sciences, University of Iceland, Reykjavík, Iceland; b Development Centre for Primary Healthcare, Iceland; c Department of Family Medicine, University of Iceland, Reykjavík, Iceland

**Keywords:** Antibiotic prescribing, children, antibiotic surveillance, primary care, respiratory infections

## Abstract

**Objective:**

To study antibiotic prescriptions among 0- to 4-year-old children before and after implementing a quality project on prudent prescribing of antibiotics in primary healthcare in the capital region of Iceland.

**Design:**

An observational, descriptive, retrospective study using quantitative methodology.

**Setting:**

Primary healthcare in the Reykjavik area with a total population of approximately 220,000.

**Subjects:**

A total of 6420 children 0–4 years of age presenting at the primary healthcare centres in the metropolitan area over three years from 2016 to 2018.

**Main outcome measures:**

Reduction of antibiotic prescriptions and change in antibiotic profile. Data on antibiotic prescriptions for children 0–4 years of age was obtained from the medical records. Out-of-hours prescriptions were not included in the database.

**Results:**

The number of prescriptions during the study period ranged from 263.6 to 289.6 prescriptions/1000 inhabitants/year. A reduction of 9% in the total number of prescriptions between 2017–2018 was observed. More than half of all prescriptions were for otitis media, followed by pneumonia and skin infections. Amoxicillin accounted for over half of all prescriptions, increasing between 2016 and 2018 by 51.3%. During this period, the prescribing of co-amoxiclav and macrolides decreased by 52.3% and 40.7%, respectively. These changes were significant in all cases, *p* < 0.0001.

**Conclusion:**

The results show an overall decrease in antibiotic prescribing concurrent with a change in the choice of antibiotics prescribed and in line with the recommendations presented in the prescribing guidelines implemented by the Primary Healthcare of the Capital Area, and consistent with the project’s goals.

**Key points**
- A substantial proportion of antibiotic prescribing can be considered inappropriate and the antibiotic prescription rate is highest in Iceland of the Nordic countries.
- After implementing guidance on the treatment of common infections together with feedback on antibiotic prescribing, a decrease in the total number of prescriptions accompanied by a shift in the antibiotic profile was observed.

## Introduction

Antibiotic-resistant bacteria pose a threat to human health, with inappropriate prescribing of antibiotics driving the evolution of resistance [1].

Studies have shown that up to 30 to 50% of antibiotic prescriptions can be considered inappropriate, mainly due to unsuitable prescribing, unfit treatment time or patients not getting clear treatment information [2]. Unnecessary and excessive use of antibiotics can have harmful consequences for patients, such as developing various kinds of allergies and asthma. This is particularly the case for children whose immune system is not mature enough to fully fight off pathogens [3,4]. A correlation between the overuse of antibiotics and alterations in gut microbiota has been observed, leading to the development of various disorders [5].

In Iceland, antibiotic resistance is relatively low compared to many other countries regarding multi-resistant bacteria such as MRSA and ESBL-producing bacteria [6]. However, results of routine antimicrobial susceptibility testing at Landspitali – The National University Hospital of Iceland (LUH) indicate growing resistance in common bacteria to prevalent antibiotics [7]. At the same time, it has diminished in the other Nordic countries [8].

The rate of antibiotic prescribing in Iceland was high during the period 2009–2018 compared to other Nordic countries and somewhat below the midrange in a European context. Besides, broad-spectrum antibiotics are more often prescribed [9]. According to a study on antibiotic prescriptions in out-of-hours primary care in Iceland, co-amoxiclav is by far the most prescribed antibiotic for respiratory tract infections in paediatric encounters [10].

Most antibiotic prescriptions are issued by general practitioners (GPs) with respiratory infections accounting for more than half of the prescriptions. Acute otitis media (AOM) is the most frequent indication [11]. AOM is a very widespread infectious condition in children [12] and the most common indication for antibiotic prescriptions for children, both in Iceland and in other Western countries, with pneumococci as the most prevalent cause of bacterial infections [13–16]. It is estimated that mortality caused by pneumococcal infections is 0.7–1 million deaths annually among children aged <5 years [17]. Additionally, it is believed that children’s use of antibiotics has a significant effect on increasing pneumococcal resistance [18].

Since 2011 pneumococcal vaccination has been a part of routine vaccinations for children in Iceland to prevent invasive infections as well as to counteract the bacterial resistance due to the emergence of multi-resistant strains [19,20]. After the implementation of pneumococcal vaccination in Iceland, hospital visits due to AOM and pneumonia have been significantly reduced [20]. Simultaneously, a reduction of antimicrobial prescribing for young children was observed, mostly due to fewer episodes of AOM [21]. The Directorate of Health has reported a 9% reduction in antibiotic prescriptions for children 0-4 years of age in Iceland from 2014–2018 [22].

The Primary Health Care of the Capital Area (PHCA) issued guidance to promote rational use of antibiotics among GPs in to contain the overall prescribing of antibiotics, especially broad-spectrum antibiotics, as there is an indication of growing resistance in common bacteria in Iceland. This included implementing new prescribing guidelines in spring 2017 [23] with the Swedish antibiotic stewardship programme STRAMA set as a model [8], taking into account differences in susceptibility of some common bacteria in Iceland compared to Sweden, leading to different recommendations when required. This was followed by meetings with GPs at the healthcare centres in the capital area. The objectives set were to reduce prescriptions of broad-spectrum antibiotics, such as co-amoxiclav and azithromycin, to reduce the number of prescriptions issued for 0–4 years old children by 10% per year, as well as to reduce the number of antibiotic prescriptions in general.

This study aimed to examine changes in antibiotic prescriptions for young children issued by GPs in primary healthcare centres during ordinary hours of work in the Reykjavik area after initiating the quality project in 2017. The PHCA initiated this project in cooperation with the Department of Clinical Microbiology LUH and the Chief Epidemiologist. Out-of-hours on-call services of GPs are beyond the jurisdiction of PHCA.

## Material and methods

### Design and setting

A descriptive retrospective data study was performed using a quantitative methodology involving data collection and statistical analysis. It was a population-based observational study. In May 2017, the PHCA antibiotic stewardship team visited all the healthcare centres under its umbrella and presented the GPs’ prescribing pattern at each healthcare centre. Data on issued prescriptions extracted from the PHCA medical records database was presented and the newly approved prescribing guidelines on antibiotics for common infections in primary care were discussed. The PHCA with 136 GPs comprising the medical staff includes 15 out of 19 primary healthcare centres in the Reykjavik area, with a population of just over 200.000. However, the PHCA does not provide an on-call service outside regular working hours.

### Study population

The study population consisted of all children aged 0–4 years (both years included) who had been prescribed antibiotics from ATC class J01 by the PHCA GPs over three years during opening hours (08-18), from 1st January 2016 to 31st December 2018. Out-of-hours prescribing is not included in the medical records database. The children are included in the study until the age of five. As soon as they reach that age, they are excluded from the study population. Each child could be prescribed antibiotics more than once during the study period so that they could repeatedly appear in the same year as well as in other years.

### Data collection

In this study, electronically issued prescriptions from the PHCA medical records database were extracted for all antibiotics in the ATC class J01 for children aged 0–4 years from 1st January 2016 to 31st December 2018. Each person has a unique personal ID number, which is always entered whenever any patient information is recorded in the medical records database. The baseline data collected in the study included age, sex, ICD-10 diagnoses at the time of prescribing, antibiotic class, the number of prescriptions, and the date of the issue.

After having extracted the data, it was encrypted and collected in a study database. Thus, each child was assigned a unique encryption ID number, so the personal identity of the child would not be revealed during the processing of the data set.

### Outcome measures

The study’s numerical variables comprised the children’s age, the date of issue of the antibiotic prescriptions and their ID number. The sex of the child, the antibiotic class, and the ICD-10 diagnoses underlying the prescriptions were the categorical variables of the study. The number of antibiotic prescriptions was defined as the response variable that was evaluated for the explanatory variables: sex, age, the date of issue, antibiotic class and ICD-10 diagnoses underlying the prescription.

The categorical variables sex and age were assessed regarding; numbers of prescriptions, which antibiotic classes were prescribed and prescription indication.

### Statistical analysis

Systemic and statistical methods were used in the processing and interpretation of the data in the R-studio. Chi-square test was used to evaluate statistically significant association in the number of antibiotic prescriptions by different categorical variables. Quasi-Poisson distribution was used to evaluate year-on-year antibiotic prescription changes. Significance was defined with 95% confidence (*p* value < 0,05).

## Results

The study population consisted of 6420 children in total over these three years. The total number of antibiotic prescriptions over the study period was 11,583. In the three years from 2016 to 2018, 22.6% of the total GP visits in the group of 0–4 years old resulted in an antibiotic prescription. The number of prescriptions increased by 2.3% between the years 2016 and 2017, that is, from 282.9 to 289.6 prescriptions/1000 inhabitants/year, although the difference was not statistically significant (*p* = 0.98). However, there was a 9% decrease in antibiotic prescriptions between 2017 (289.6) and 2018 (263.6) coinciding with the implementation of the new guidance on treatments of common infections in primary care (*p* = 1.6*10^−7^). Thus, the number of prescriptions during the study period ranged from 263.6 to 289.6 prescriptions/1000 inhabitants/year. More than half of the prescriptions were issued for boys or 52.5%, and most of the antibiotic prescriptions were intended for the age group 1-2 years old. Followed by children 2- to 3-year-old, then 0–1- and 3- to 4-year-old children. The fewest antibiotic prescriptions were for the 4-year-old children.

Table 1 provides an overview of the number of antibiotic prescriptions during the study period in which antibiotics were prescribed based on the ICD-10 diagnosis. The table only covers the following diagnoses; cystitis, otitis media, pneumonia, sinusitis, streptococcal infection in the throat and tonsillitis. Other ICD-10 diagnoses were collectively put under the category ‘Other.’

**Table 1.** The number of prescriptions for each antibiotic group as well as the underlying ICD-10 diagnoses for each study year.

	Diagnoses																				
	Cystitis			Otitis media			Pneumonia			Sinusitis			Streptococcal infection in the throat			Tonsillitis			Other ICD-10 diagnoses		
	Study period																				
	2016	2017	2018	2016	2017	2018	2016	2017	2018	2016	2017	2018	2016	2017	2018	2016	2017	2018	2016	2017	2018
Antibiotic categories (ATC code)	Number of antibiotic prescriptions																				
Tetracyclines (J01AA)				1																	
Penicillin with extended spectrum (J01CA)	7	8	9	1082	1564	1621	66	147	153	37	86	57	36	24	15	63	66	39	287	369	386
Beta-lactamase sensitive penicillin (J01CE)				39	8	18	6	5	1			3	138	82	47	169	112	76	99	59	56
Beta-lactamase-resistant penicillin (J01CF)																			1		3
Combinations of penicillin, incl. beta-lactamase inhibitors (J01CR)	3	2	2	711	442	357	124	64	28	46	31	6	20	5	1	25	15	6	281	180	153
First-generation cephalosporins (J01DB)	10	20	20	202	169	123	13	14	15	6	2		15	9	1	23	27	15	227	265	210
Trimethoprim and derivatives (J01EA)	17	14	10	1	2														13	18	11
Combinations of sulphonamides and trimethoprim, incl. derivatives (J01EE)	9	22	19	36	24	25	2		1										33	30	22
Macrolides (J01FA)				41	35	18	39	33	13	1	1		3	2		10	2	1	53	39	33
Fluoroquinolones (J01MA)																			2	1	1
Nitrofurantoin derivatives (J01XE)	2	2	1																3	3	1
All diagnosis per year	48	68	61	2113	2244	2162	250	263	211	90	120	66	212	122	64	290	222	137	999	964	876

### Antibiotics prescribed

**Figure 1.**
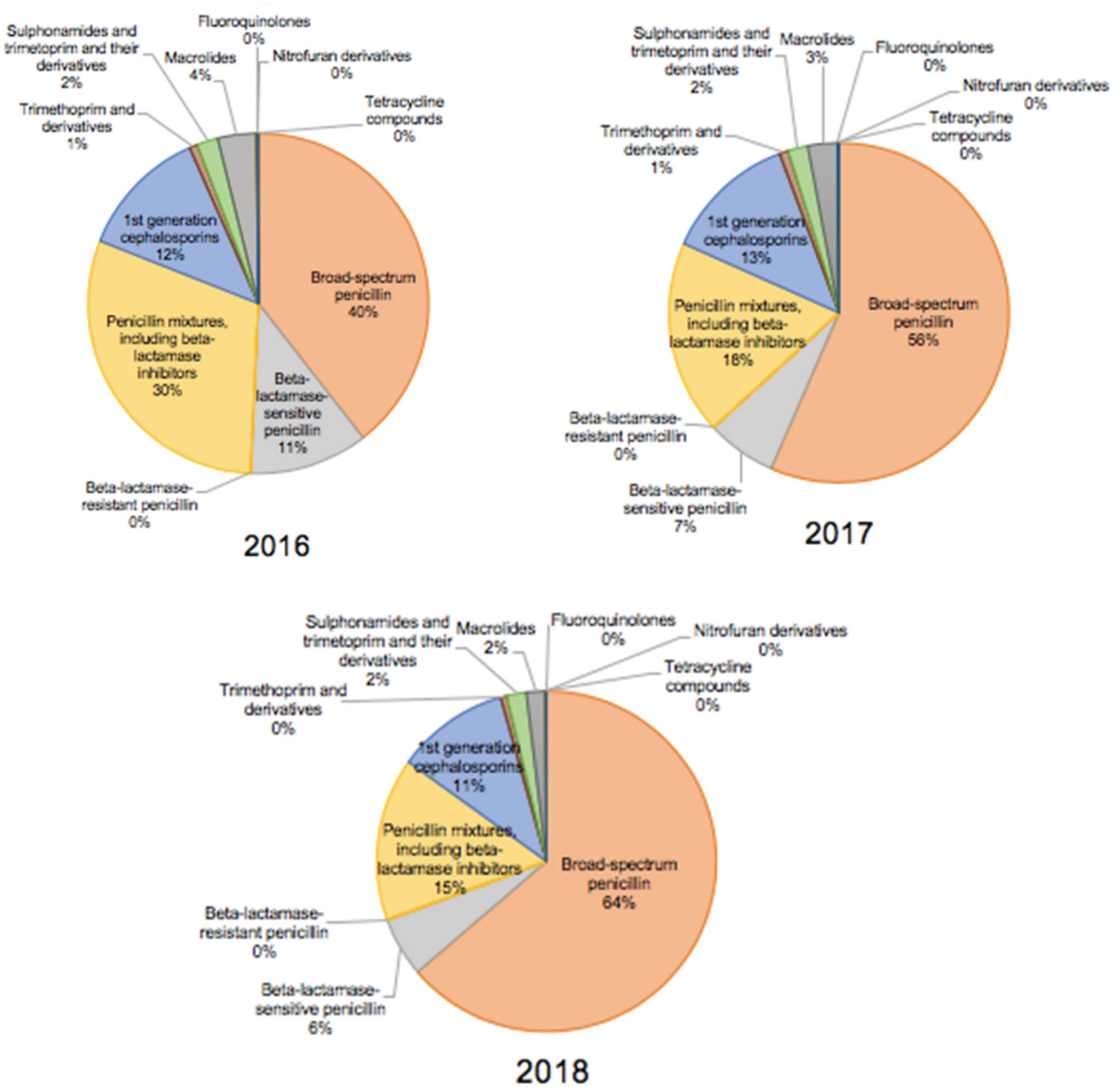
Percentage of antibiotic prescriptions by antibiotic categories for each study year.

**Figure 2.**
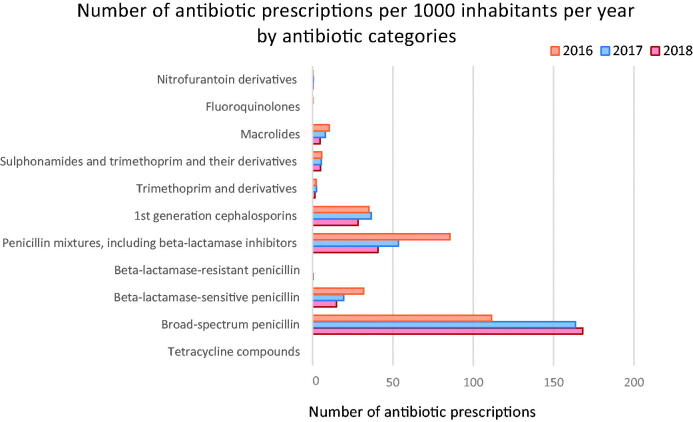
Overview of the number of antibiotic prescriptions per 1000 0–4 years old inhabitants per year by antibiotic categories during the study period.

The children’s antibiotic prescriptions were classified into 11 ATC groups. Extended-spectrum penicillin (J01CA), almost solely amoxicillin, comprised more than half of all the antibiotic prescriptions and increased significantly over the three years (*p* = 2.2*10^−16^). Their number increased from 40.0% of the total antibiotic prescriptions in 2016 to 64.0% in 2018 (Figure 1), that is, from 111 to 168 prescriptions per 1000 inhabitants per year (Figure 2). Co-amoxiclav which belongs to the ATC class J01CR was the second most prescribed antibiotic, followed by the first-generation cephalosporins (J01DB) (Figures 1–2). The number of macrolide (J01FA) prescriptions, mostly comprising azithromycin, decreased significantly over the study period (*p* = 1.6*10^−7^) or about 40.7% in 2018, compared to 2017 after the implementation of the new guidance, as well as the prescriptions of co-amoxiclav which decreased by 52.3% (*p* = 2.2*10^−16^) at the same time.

### Diagnoses

**Figure 3.**
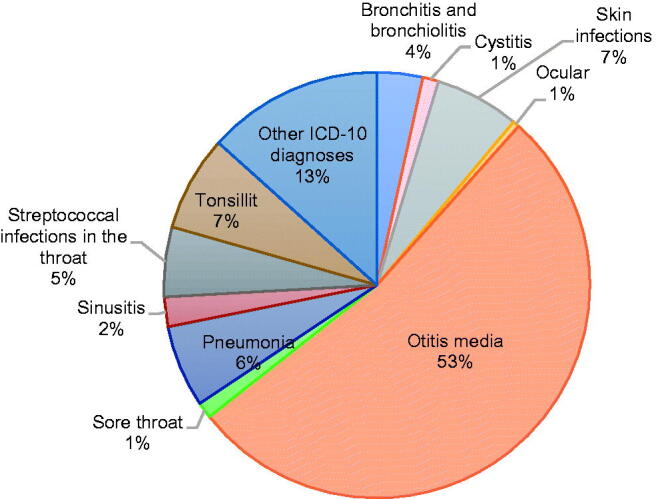
Overview of the percentage of antibiotic prescriptions associated with each diagnosis over the entire study period.

The ICD-10 diagnoses that led to antibiotic treatment in the children during the study period were classified into 11 categories. Most of the diagnoses are classified as respiratory infections, apart from skin infections, cystitis, ocular infections, and infections that fall collectively under the ill-defined group ‘Other infections.’ The relevant diagnoses are listed in Supplementary Table 1. Figure 3 gives an overview of the percentage of antibiotic prescriptions that were associated with each diagnosis over the entire study period. More than half of all prescriptions were for otitis media (53%), followed by pneumonia and skin infections.

### Otitis media and pneumonia

More than half of all the prescriptions were for otitis media, followed by pneumonia (Figure 3 and Table 1). There was a significant increase in the use of extended-spectrum penicillin for otitis media (*p* = 2.2*10^−16^) and pneumonia (*p* = 3.9*10^−9^).

### Tonsillitis and streptococcal infection

In most cases, phenoxymethylpenicillin was the drug prescribed for tonsillitis and streptococcal infections and the number of prescriptions decreased significantly for both tonsillitis (*p* = 9.4*10^−9^) and streptococcal infections (*p* = 5.2*10^−11^) over the study period (Figure 3 and Table 1).

### Sinusitis

Amoxicillin and co-amoxiclav were the most prescribed drugs for sinusitis. The prescription pattern reveals that the number of prescriptions of co-amoxiclav declined over the study period. However, the proportional use of amoxicillin increased significantly (Figure 3 and Table 1).

### Cystitis

Most antibiotic prescriptions for cystitis were from the following groups: first-generation cephalosporins, trimethoprim, and combination of sulphonamides and trimethoprim (Figure 3 and Table 1).

## Discussion

### Statement of principal findings

During the study period, we observed a pronounced reduction of antibiotic prescriptions for children and a shift in the antibiotic profile with highly significant changes in the prescription of certain antibiotics. These changes occurred in the wake of the implementation of a quality project on prudent prescriptions of antibiotics.

### Strengths and weaknesses of the study

Data in our study is retrieved from a comprehensive medical records database of 15 primary healthcare centres in the Reykjavík metropolitan area, lending strength to its generalisability. The data covers three years during the implementation of guidance to promote rational use of antibiotics among GPs, that is, the state of antimicrobial prescribing in the year before the implementation 2016, the year of the implementation 2017 and the year after the implementation 2018. Nevertheless, there are some limitations in our study. Using issued prescriptions as a measurement of antibiotic consumption may be slightly inaccurate due to non-redeemed prescriptions. An overall reduction of antibiotic prescribing was observed for the whole country over a five-year period from 2014–2018 which could lead to some confounding when estimating the corresponding reduction between 2017 and 2018 in the metropolitan area. We do not have information on the prescription rate for the most common diseases which can be considered a limitation. An important limitation is that the database did not cover out-of-hours primary care services. The study is an observational study, not a causal-comparative research, and thus, we are unable to prove a causal relationship between the implementation and the changes observed. Besides, one can never be certain that observational data are not hiding a confounding variable, rendering the observational nature of the study a limitation.

### Findings in relation to other studies

Antibiotic prescriptions were most common in the second year of age and fewest in the four-year-old children. A Danish study of 0- to 6-year-old children found antibiotic use to be highest among one-year-old children, which is consistent with our results, but then it decreased with increasing age [24]. Furthermore, an Icelandic study shows similar results, with antibiotic use being highest among the youngest children [25]. A possible explanation is that children’s susceptibility to infections increases considerably after their mothers’ antibodies disappear and before their antibodies are produced in sufficient quantities. This process usually occurs just before they turn one-year old [3]. Research also supports that children who attend day-care centres are at a higher risk of getting infections than children who stay at home. They have more doctor visits and are therefore more likely to be prescribed antibiotics [26,27].

### Most common diagnoses and changed prescribing of antibiotics

Acute otitis media (AOM) was the most common reason for antibiotic prescriptions, as expected [28], followed by pneumonia and skin infections. Only a reduction of 2.7% was observed in antibiotic prescriptions for AOM between the years 2017–2018. The data neither reveals the incidence of AOM during the study period nor how often AOM was either treated or not treated with antibiotics. In this context, a Swedish study can be considered where the incidence of AOM was 33.2–35.8% in 0–2 years old children and 31.9–32.9% in the 3–7 years old group [29]. In 2009, a general infant pneumococcal vaccination was introduced in Sweden. A study comparing the prevaccination period of 2005–2008 to 2014 found that in children 0–4 years of age, a reduction of 46.4% was observed for acute otitis media with an ICD code specifying it as a bacterial infection [30]. Likewise, a reduction in the incidence of AOM was observed after implementing a pneumococcal conjugate vaccine into the routine childhood vaccination programme in Iceland in 2011 [31]. A study on the impact of the 10-valent pneumococcal conjugate vaccine on antimicrobial prescriptions in young children revealed the greatest reduction in all-cause antimicrobial consumption in the first vaccine-eligible cohort in 2011 [21]. Thereafter, it changed very little, rendering unlikely the possibility of a notable influence of the vaccination programme starting in 2011 on the changes observed in our study between 2016 and 2018. A distinct and noticeable reduction was observed in the prescribing of co-amoxiclav, macrolides and cephalosporins, and simultaneously an increase in amoxicillin prescriptions during the study period, indicating a shift in the choice of antimicrobial therapy in AOM. Similar changes were observed for the antibiotic prescriptions for pneumonia. These changes were in accordance with the recommendations for the selection of appropriate antimicrobial therapy for common infections in primary care presented in the new prescribing guidelines in 2017, where amoxicillin was recommended as a first-line antibiotic for AOM and pneumonia [23]. An impressive reduction was observed in all antibiotic prescriptions for streptococcal infection in the throat and for tonsillitis, which raises the question if these changes might have been caused by a change in the incidence of streptococcal throat infections. Phenoxymethylpenicillin was the most commonly prescribed antibiotic for these diagnoses in accordance with the recommendations from 2017, and the fact that the resistance rate in *Streptococcus pyogenes* for penicillin in Iceland has been determined to be 0% [7].

### Reduced prescribing of antibiotics

A reduction of 9% in the total prescribing of antibiotics was observed, and at the end of the study period, it amounted to 264 prescription per 1000 inhabitants per year. Our data neither include prescriptions issued during out-of-hours services in primary care in the capital area, primary care outside the capital area nor data from specialists’ clinics. The total sale of antibiotics for children 0–4 years of age in outpatient care in Sweden was 320 prescriptions per 1000 inhabitants per year in 2018 [32]. According to the Director of Health in Iceland, the total sale of antibiotics in outpatient service to children 0–4 years of age was 1025 prescriptions per 1000 inhabitants per year and for all age groups it was 668 prescriptions/1000 inhabitants/year [22]. In the capital area, 264/1025 or only 25% of prescriptions to children are issued by GPs in the PHCA during normal working hours. Of great concern in Iceland is the high proportion (30%) of *Streptococcus pneumoniae* strains with intermediate resistance to penicillin, and considerable resistance to erythromycin (39%) and tetracyclines (27%), according to results from routine antimicrobial susceptibility testing at LUH [7]. *Streptococcus pneumoniae* is one of the most common bacterial causes of AOM, and the connection between the use of antibiotics and resistance is well known [25]. Therefore, it is critical to reduce the number of antibiotic prescriptions in Iceland, not least for children, to restrain further development of resistance to antibiotics in Iceland [33].

Although for a long time the Chief Epidemiologist and other official stakeholders have urged doctors to prescribe antibiotics more wisely, it may be that the lack of a structured programme to reduce antimicrobial prescribing and old habits have contributed to keeping the antibiotic prescriptions rate higher in Iceland compared to other Nordic countries. However, we do not have any clear-cut data to substantiate this. In 2020, an application was added to the medical-records software allowing the prescribers to view their prescriptions in comparison with colleagues at the healthcare centre and the prescribing at the PHCA in general. Studies have shown how key issues in educational interventions and individually targeted communication can improve antimicrobial prescribing [34,35].

During the 25 years of the Swedish STRAMA programme, antibiotic prescriptions have declined dramatically in Sweden, especially for children, and the changes in antibiotic prescriptions for children observed in our study are an encouragement to continue this quality project to urge a more prudent use of antibiotics in the Icelandic health service.

### Meaning of the study

This study examined changes in antibiotic prescriptions for young children issued by GPs in primary healthcare centres during ordinary hours of work in the Reykjavik metropolitan area after initiating the quality project on prudent use of antibiotics in 2017. The changes observed should encourage primary care in the whole country to continue with projects on quality improvement in antibiotic prescribing. The first steps have been taken in this respect. Accordingly, the main practical contribution of the present research is that it demonstrates how quality programmes within primary care settings can be an efficient approach for changing the prescribing habits of GPs. In our setting, it was implemented by visiting all the healthcare centres, presenting the GPs with their prescription patterns and promoting the prescribing guidelines as a discussion platform for improvement. Future studies will have to show if similar methods can be used to encourage prudent prescriptions of other drug classes such as opioids and psycholeptics.

## Conclusion

We observed a marked change in the choice of antibiotics prescribed, in line with the prescribing guidelines’ recommendations. Concurrently, a distinct overall decrease in the prescribing of antibiotics for children 0–4 years of age was observed over the period following the implementation of the guidelines.

## Supplementary Material

Supplemental Material
