# Supplementary material for: Encouraging rational antibiotic prescribing behaviour in primary care – prescribing practice among children aged 0–4 years 2016–2018: an observational study
Source: Scand J Prim Health Care. 2021 Aug 4;39(3):373–81. doi: 10.1080/02813432.2021.1958506 (PMC8475099; doi:10.1080/02813432.2021.1958506)
Supplement: Supplemental Material [file IPRI_A_1958506_SM4553.docx]

Supplementary table 1

| **Disease** | **ICD-10 code** |
| --- | --- |
| Streptococcal throat infections | A38 + B95-B95.5 + J02.0 |
| Tonsillitis | J03-J03.9 |
| Conjunctivitis | H00-H10.9 |
| Otitis media | H65-H67.0+ |
| Sinusitis | J01-J01.9 + J32-J32.9 |
| Acute pharyngitis, unspecified | J02.9 |
| Bacterial pneumonia | J15-J18.9 |
| Bronchitis and bronchiolitis | J20-J20.9 + J40 +J21-J21.9 + J44.8 |
| Cystitis | N30.0-N39.0 |
| Dermatological infections | L03-L03.9 + L08.0-L08.9 + L02-L02.9 + L01-L01.0 + B01-B01.9 |
| Other diagnoses | A09-A31.9, A46-A74.0+, B08.1+-B35.4, H20.0-H60.9, H72-H93.9, I88.9-I89.1, J00, J04-J11.1, J22-J35.1, J45.9, K04-K59.0, L04, L13.0-L98.4, M86, N10-N13.7, N47-N89.8, Nanda, P23.6-P39.4, Q33.0, R01-R68.8, S00.8-S91.2, T14.0-T88.0, W54.9, X51, Z03-Z76.9 |
